# Leptin levels and leptin receptor polymorphism frequency in healthy populations

**DOI:** 10.1186/1750-9378-4-S1-S13

**Published:** 2009-02-10

**Authors:** Camille C Ragin, Cher Dallal, Michael Okobia, Francesmary Modugno, Jiangying Chen, Seymour Garte, Emanuela Taioli

**Affiliations:** 1 Department of Epidemiology, University of Pittsburgh Graduate School of Public Health, Pittsburgh, USA; 2 University of Pittsburgh Cancer Institute, Pittsburgh, USA; 3 Department of Surgery, College of Medical Sciences, University of Benin, Benin City, Nigeria; 4 Department of Environment and Occupational Health, University of Pittsburgh Graduate School of Public Health, Pittsburgh, USA; 5 Guangzhou Red Cross Hospital, Guangdong, PR China; 6 Department of Epidemiology and Biostatistics, Downstate School of Public Health, State University of New York, USA

## Abstract

**Background:**

The leptin receptor gene (*LEPR) *polymorphism Q223R is one of the most common in the general population, and is thought to be associated with an impaired signaling capacity of the leptin receptor and with higher mean circulating levels of leptin. Leptin is a hormone primarily produced in adipose tissue. Increased levels of leptin have been positively correlated with obesity. We have determined the frequency of the leptin receptor polymorphism (*LEPR *Q223R) in healthy populations from various ethnic groups, and compared plasma leptin levels across the *LEPR *Q223R polymorphism in healthy African-Caribbean and Caucasian women.

**Results:**

The study population consists of 1,418 healthy subjects from various ethnic groups. The *LEPR *Q223R homozygous variant was observed overall in 19% of subjects (n = 1,418), with significant differences based on self reported ethnicity: the proportion of subjects with the homozygous variant was lower in Caucasians (14%, n = 883) than in African-Caribbean (n = 194), African-American (n = 36) and Asian/other ethnic groups (n = 26), (35%, 33% and 34.6% respectively); the frequency in Africans (20%), was similar to the overall study population. The mean ± standard deviation (SD), circulating leptin levels for African-Caribbean women was 44.7 ± 31.4 ng/ml, while for Caucasian women the mean was 42.4 ± 34.8 ng/ml. Adjusted circulating leptin levels in post-menopausal Caucasian women who were *LEPR *Q223R homozygous variant were marginally statistically significantly higher than in women with the wild-type genotype (p = 0.098). No significant differences in leptin levels by genotype were observed for African-Caribbean women, (heterozygous: p = 0.765, homozygous variant: p = 0.485).

**Conclusion:**

These findings suggest an association between mean circulating leptin levels and the *LEPR *Q223R genotype among post-menopausal Caucasian women.

## Introduction

Leptin, a product of the ob gene, is a biologically active polypeptide (16 kDa) primarily produced in adipose tissue [1]. Increased levels of leptin have been shown to correlate with obesity [2], a known risk factor for postmenopausal breast cancer [3-7]. Some studies have reported that leptin stimulates the proliferation of normal and malignant breast epithelial cells [8] and others have suggested that leptin levels may be associated with breast cancer [9,10] as well as cancer at other sites [11]. Leptin binds to the human leptin receptor, a cytokine receptor that promotes gene transcription via activating signal transduction pathways [12]. The function of leptin is mediated through the leptin receptor, and both leptin and its receptor are involved in the homeostatic control of appetite, weight, metabolism and reproductive functions in women [13]. A number of polymorphisms have been reported in the human *LEPR *gene [14]. The *LEPR *polymorphism Q223R is one of the most common and is thought to be associated with an impaired signaling capacity of the leptin receptor; this polymorphism has been associated with higher mean circulating levels of leptin [15,16].

We have previously conducted a meta-analysis (n = 18 studies) to examine the frequencies of *LEPR *polymorphisms in the general population [14]. From this analysis of the literature, it became apparent that only one study included subjects of African ancestry; however, this study sample was predominantly Caucasian [17]. Although research has been conducted on circulating leptin levels among healthy populations of women including women of African descent [18-24], few studies have evaluated mean circulating leptin levels according to the *LEPR *Q223R genotype [15,16,25] among healthy populations of women. Furthermore, no previous study has evaluated leptin levels by the *LEPR *Q223R polymorphism among African-Caribbean women.

In this study, we have determined the frequency of leptin receptor polymorphism (*LEPR *Q223R) in healthy populations from various ethnic groups (African, African-American, African-Caribbean, Caucasian, Asian/and other ethnic groups). We have also compared the *LEPR *Q223R polymorphism with plasma leptin levels in healthy African-Caribbean and Caucasian women.

## Materials and methods

### Study population

The study population consists of 1,418 healthy subjects (883 Caucasians, 279 Africans, 36 African-Americans, 194 African-Caribbean, 26 Asian/other ethnic groups). Caucasian subjects were recruited as part of a study on hormonal determinants of mammographic density [26] as well as a study of hormones, bone density and genetic markers of susceptibility [27]; African-American and African subjects were part of a study on ethnic differences in polymorphisms of genes involved in tobacco metabolism [28]; African-Caribbean subjects were recruited as part of a study on cervical and oral HPV infection [29]. Ethnicity for all subjects was self-reported. Nineteen of the 26 Asian subjects were recruited from the Caribbean but self-reported their ethnicity as East Indian.

Women had no history of hormone replacement therapy (HRT) as determined from questionnaire data. There were 6 African-Caribbean women and one Asian who reported ever HRT users and for 36 African-Caribbean and two Asian women HRT use was not reported. Menopausal status was determined by using a self reported questionnaire on whether their menses has stopped and/or date at the last menstrual cycle. If the date of last menstruation was prior to six months from recruitment, women were considered post-menopausal. One subject who reported that her menses stopped 3 months before recruitment was included in the post-menopausal group. Twenty-nine 29 African-Caribbean women responses were missing therefore were unable to define their menopausal status.

### Leptin levels

Nonfasting plasma leptin levels were determined for a subpopulation of women (N = 574) using a commercially available leptin ELISA kit (Biosource International, Camarilli, CA, USA). Batches were performed using 96 well plates which included ~38 samples, 8 leptin standards and quality controls. Forty blinded duplicates were included as a means of assessing the reliability of the assay. Each sample was run in duplicate within the same plate and a coefficient of variation (CV) was calculated. All CV's greater than 15% were repeated. The average of the two leptin measurements was used for this analysis.

### *LEPR *Q223R genotyping

DNA was extracted from blood samples and the allelic frequency of *LEPR *Q223R was evaluated for all subjects by polymerase chain reaction (PCR) of the extracted DNA, followed by *MspI *restriction enzyme digestion. The PCR primers and cycling conditions used have been previously published [30]. Briefly, amplification of the 80bp *LEPR *PCR product was visualized following PCR amplification and agarose gel electrophoreses. The amplified product was then subjected to *MspI *restriction enzyme digestion at 37°C overnight. Samples from subjects with the wild-type gene yielded an 80bp DNA band after *MspI *digestion. A heterozygous subject sample was indicated by 80 bp, 58 bp and 22 bp PCR products while a homozygous variant was indicated by 8 bp and 22 bp PCR products only. Twenty samples were randomly chosen and repeated in blind fashion to assess the reproducibility of the method.

### Statistical analysis

Statistical analyses were performed using STATA (version SE 10.0) software (StataCorp. LP, College Station, TX). Pearson's and the likelihood-ratio chi-squared test were used to assess Hardy-Weinberg equilibrium. Leptin levels were square root transformed in order to obtain a normal distribution for parametric tests. The independent effect of body mass index, age, menopausal status and *LEPR *genotype on leptin levels by ethnic group was assessed using a two-way analysis of variance (ANOVA).

## Results

### *LEPR *Q223R polymorphism distribution

The *LEPR *Q223R genotype frequency based on ethnicity is summarized in Table 1. For the overall study population, the frequency of the heterozygous (GA) and homozygous (AA) variants was 50% and 18.97% respectively and significant differences were observed based on ethnicity (p < 0.0001).

**Table 1:** Distribution of the *LEPR *Q223R genotype across ethnic groups.

***LEPR *Q223R**	**African** **N (%)**	**African-American N (%)**	**African-Caribbean N (%)**	**Caucasian N (%)**	**Asian/Other N (%)**	**TOTAL** **N (%)**
GG	69 (24.73)	6 (16.67)	26 (13.40)	335 (37.94)	4 (15.38)	440 (31.03)
GA	155 (55.56)	18 (50.00)	100 (51.55)	423 (47.90)	13 (50.00)	709 (50.00)
AA	55 (19.71)	12 (33.33)	68 (35.05)	125 (14.16)	9 (34.62)	269 (18.97)

Total	279 (100)	36 (100)	194 (100)	883 (100)	26 (100)	1,418 (100)

Higher frequencies of the AA variant was observed among African-Caribbean 35.1%), African-Americans (33.3%) and Asian/Other subjects (34.6%) as compared to the overall study population. In contrast, Caucasian subjects had the lowest frequency of the AA variant (14.2%); the frequency of the AA variant in Africans was similar to the overall study population.

We observed no violation of Hardy Hardy-Weinberg equilibrium for either the overall study population or after stratification by ethnic groups (p > 0.1). The highest proportion of subjects with the heterozygous (GA) variant was observed in African subjects (55.7%), while Caucasian subjects had the lowest frequency of the GA variant (47.9%).

### Circulating plasma leptin levels in females

Plasma leptin levels were available for 574 female subject (149 pre-menopausal and 425 post-menopausal). These included 340 Caucasians and 19 African-Americans post-menopausal women, 191 African-Caribbean women (135: pre-menopausal and 56: post-menopausal), and 24 women of Asian and other ethnic derivations (14: pre-menopausal and 10: post-menopausal). For the entire study population, we observed a mean circulating plasma leptin level of 43.5 ± 33.3 ng/ml. A summary of the mean plasma leptin levels stratified by menopausal status is shown in Table 2. Caucasian and African-American populations consisted of post-menopausal women only, with an overall mean circulating leptin level of 42.4 ± 34.8 ng/ml and 61.1 ± 26.7, respectively ng/ml. Mean age was available for all ethnic groups (African: 44.7 years, African-American: 46.5 years, Caribbean: 41.1 years, Caucasian: 50.1 years and Asian/other ethnic groups: 43.7 years), but body mass index (BMI) was not available for the Caribbean population (African: 24.7 kg/m^2^, African-American: 28.9 kg/m^2^, Caucasian: 26.2 kg/m^2 ^and Asian/other ethnic groups: 24.7 kg/m^2^). After adjusting the transformed mean leptin levels for age and BMI, the mean circulating plasma leptin level for African-Americans was marginally statistically significantly higher than Caucasians women (p = 0.07). All other ethnic groups consisted of both pre- and post-menopausal women. African-Caribbean women had a mean overall plasma leptin level of 44.7 ± 31.4 ng/ml (pre-menopausal = 41.8 ± 27.8 ng/ml vs. post-menopausal subjects = 51.7 ± 38.2 ng/ml, adjusted transformed mean comparisons: p > 0.1). The overall mean levels observed in African-Caribbean women were statistically significantly higher than Caucasian women after adjusting for age (p = 0.04); body mass index data was not available for the African-Caribbean women. For Asian and other ethnic groups, the mean overall plasma leptin level was 35.3 ± 26.3 ng/ml (pre-menopausal = 37.9 ± 30.4 ng/ml; post-menopausal = 31.6 ± 20.0 ng/ml). After adjusting for age and BMI, the mean transformed levels observed for post-menopausal Asian/other women was not statistical significantly different from that of Caucasians (p > 0.1). For pre-menopausal women we did not observe a statistically significant difference between African-Caribbean and Asian/other ethnic groups, after adjusting for age (p = 0.15).

**Table 2:** Mean circulating leptin levels according to ethnicity and menopausal status.

	**Mean plasma Leptin levels ± SD**				
**Race/Ethnicity**	**Pre-menopausal**	**N**	**Post-menopausal**	**N**	**p-value**

**African-American (N = 19)**	-	-	61.1 ng/ml ± 26.7	19	0.074*
**African-Caribbean (N = 191)**	41.8 ng/ml ± 27.8	135	51.7 ng/ml ± 38.2	56	0.039**
**Caucasian (N = 340)**	-	-	42.4 ng/ml ± 34.8	340	
**Asian/Other (N = 24)**	37.9 ng/ml ± 30.4	14	31.6 ng/ml ± 20.0	10	0.427*

The p-values presented are for post-menopausal women, calculated with Caucasians as the reference group and *adjusted for body mass index and age; **adjusted for age only (BMI was not available fore these subjects). SD = standard deviation, N = number.

### Circulating leptin levels and *LEPR *Q223R genotype

**Figure 1:**
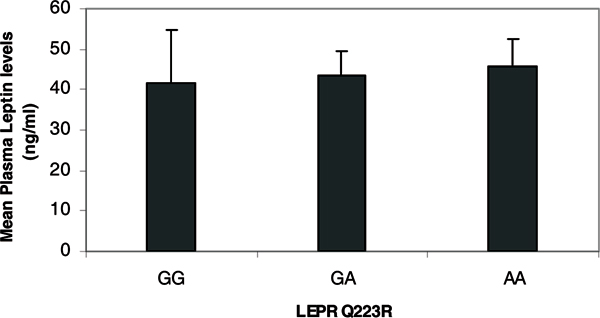
Mean circulating leptin levels according to *LEPR *Q223R genotype in African-Caribbean women.

**Figure 2:**
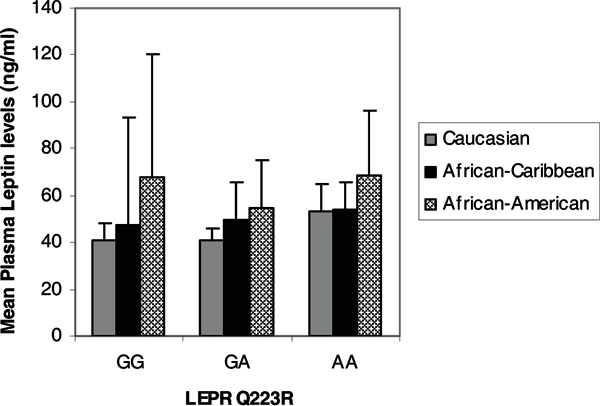
Mean circulating leptin levels in post-menopausal women according to ethnicity and *LEPR *Q223R genotype.

Figure 1 shows the mean circulating leptin levels in African-Caribbean women stratified by *LEPR *Q223R genotype. Comparisons of the transformed mean circulating leptin levels stratified by *LEPR *Q223R genotype was accomplished after adjusting for age and menopausal status (BMI data was not available for these subjects). For subjects with the wild-type (GG) genotype the mean circulating leptin levels were 43.1 ± 35.6 ng/ml, while the heterozygous (GA) and homozygous (AA) variants had mean leptin levels of 44.9 ± 32.2 ng/ml and 44.4 ± 28.7 ng/ml, respectively. These mean plasma leptin levels were not statistically significantly different from each other (adjusted p > 0.1). There were no statistically significant differences in mean leptin levels across the *LEPR*Q223R genotype variants among pre-menopausal African-Caribbean women (GG: 41.5 ± 31 ng/ml, GA: 42.9 ± 26.9 ng/ml, AA: 39.1 ± 28.0 ng/ml, adjusted p > 0.1). In post-menopausal women mean leptin levels were higher for the variant genotypes, but they were not statistically significantly different (GG: 47.5 ± 49.4 ng/ml, GA: 50.9 ± 44.3 ng/ml, AA: 53.7 ± 28.0 ng/ml, adjusted p > 0.1) (Figure 2).

Leptin levels were compared in post-menopausal African-Caribbean women and post-menopausal Caucasian and African-American women (Figure 2); in general, African-Americans had higher leptin levels, while Caucasian women had lower leptin levels. After adjusting for age and BMI, post-menopausal Caucasian women carrying the homozygous (AA) variant genotype had marginally statistically significantly higher leptin levels than women with the wild-type genotype (52.1 ± 45.4 ng/ml vs. 40.2 ± 32.9 ng/ml, adjusted p = 0.056). There was no statistically significant difference in leptin levels between Caucasian subjects with the heterozygous and wild-type variants (adjusted p > 0.1). Although higher leptin levels were observed among African Americans there was no statistically significant difference observed when stratified by genotype; however, there were only 19 African-American subjects (adjusted p > 0.1).

## Discussion

To our knowledge, we have for the first time described the frequency of the *LEPR *Q223R variant in healthy populations of various ethnic groups. We have also compared the genotype frequency in a subset of female subjects with their mean circulating leptin levels. We observe statistically significant differences in genotype frequency and mean circulating leptin levels based on ethnicity. African-Caribbean and African-American subjects had higher frequencies of the AA homozygous variant compared to Caucasian and African subjects. Since ethnicity was self-reported, it is difficult to evaluate the degree and type of admixture present in the populations of African descent (African-American and African-Caribbean). The analysis of the available African populations present in our study indicates that there is a great variability in the *LEPR *homozygous variant frequency in Africa, with Nigerian subjects having a higher frequency (21%) than subjects from Mali (12%). It is possible that the geographic areas from which the African populations were taken during the slave trades could influence the variations in the *LEPR *gene frequency in populations of African descent. For example, historical data seem to indicate that a higher proportion of slaves were taken to the US and Caribbean from Nigeria rather than Mali [31].

Among post-menopausal women, we observed significant differences in the mean plasma leptin levels between Caucasians (42.4 ng/ml) and subjects of African Ancestry (African-Americans: 61.1 ng/ml, African-Caribbeans: 51.7 ng/ml). These observed differences may be partly due to differences in overall body mass index in these populations [32]. In our study, average BMI in African-American post-menopausal women was statistically significantly higher than BMI in Caucasians (data not shown). The role of BMI in determining the differences in leptin levels between Caucasian women and African-Caribbean women since BMI data was not available. However, for the comparison between African-American and Caucasian women, we were able to adjust the analysis for both age and BMI, and still observed a significant difference in leptin levels between the two ethnic groups, which could be due to other environmental factors such as diet [33-35], or by differences in genetic factors regulating leptin levels [36].

In regards to this study, we were able to look at the influence of one main genetic polymorphism, *LEPR*, on circulating leptin levels. For all three ethnic groups, leptin levels were higher with the presence of the *LEPR *polymorphism, although the difference was not statistically significant. This seems to indicate that the functional significance of the polymorphism is similar across the three ethnic groups [15].

The strength of the present study is the availability of both genetic information and leptin levels in healthy populations of both African origin and African descent; the main limitations are: the modest number of African-American and Asian subjects, the lack of pre-menopausal women for some ethnic groups. A larger study on healthy subjects, with complete epidemiological information, and with genetic data on admixture is warranted. Simultaneous testing of multiple genetic polymorphisms in addition to the one currently studied is also desirable.

## Competing interests

The authors declare that they have no competing interests.

## Authors' contributions

CD, CR, ET, FM, MO and SG contributed the samples and corresponding data sets that were included in this analysis. CR performed the data analysis and JC performed the laboratory testing.
